# Cardiac magnetic resonance imaging and clinical prognosis in pulmonary arterial hypertension

**DOI:** 10.1590/0100-3984.2019.52.6e1

**Published:** 2019

**Authors:** Flávia Pegado Junqueira, Roberto Sasdeli Neto

**Affiliations:** 1 Department of Radiology, Delboni Auriemo - DASA, and Center for Diagnostic Imaging, Hospital Moriah, São Paulo, SP, Brazil. Email: junqueira.fp@gmail.com; 2 Center for Diagnostic Imaging, Hospital Moriah, and Department of Imaging, Hospital Israelita Albert Einstein, São Paulo, SP, Brazil

Pulmonary arterial hypertension (PAH) is one of the most important and potentially fatal alterations to the pulmonary circulation; if it goes untreated, it is associated with high morbidity, high mortality, and a poor prognosis^(1)^. An increase in pulmonary artery pressure (PAP) leads to secondary right ventricular (RV) failure^(2)^. In patients with PAH, the development of heart failure is an indicator of a poor prognosis^(3)^.

In the last decade, various studies have highlighted the importance of imaging methods other than angiography in the evaluation of pulmonary artery disease. One of the most widely used of such methods is cardiac magnetic resonance imaging (MRI).

For the evaluation of systolic function and the quantification of cavity volumes, as well as of the myocardial mass, cardiac MRI is considered the gold standard^(4)^. It has several advantages over other methods, mainly due to its noninvasive nature and its capacity to evaluate, in only one examination, morphology, RV function, left ventricular (LV) function, and tissue characteristics, as well as to provide functional information by perfusion imaging at rest, pharmacological stress testing, and flow studies.

Flow cardiac MRI studies can provide several noninvasive measurements that reflect the hemodynamics of the pulmonary arterial system. For example, curvature of the ventricular septum is strongly correlated with an RV > LV pressure gradient and is comparable to RV systolic pressure determined by catheterization^(5)^. The maximum angle of septal excursion toward the LV in ventricular systole-the interventricular septal angle (α)-also shows a strong correlation with PAP determined by invasive techniques^(6)^.

The mean PAP and pulmonary vascular resistance can also be estimated by cardiac MRI with regression equations^(7)^. The mean PAP estimated thusly has a sensitivity of 87% and a specificity of 90% for the diagnosis of PAP > 32 mmHg^(8)^. Pulmonary artery flow velocity has also been shown to correlate with the mean PAP.

Other variables evaluated in PAH include: the relative change in area; increase in the maximum peak velocity; the time to maximum velocity; the maximum change in flow at ejection time; increase in the oscillatory shear index; increase in the shear interval; the transpulmonary gradient in the pulmonary artery; transmitral flow; myocardial tissue velocity; left atrial volume; and left atrial flow^(8)^.

In addition to all the information provided above, cardiac MRI allows prognostic factors to be estimated and risk to be stratified. For example, delayed myocardial enhancement volume correlates with RV mass, RV volume, RV dysfunction, RV remodeling, and septal curvature, indicating a poorer prognosis^(5)^.

In this issue of **Radiologia Brasileira**, readers will find an interesting article authored by Mello et al.^(9)^, who studied the relationship between the right atrium area and the RV ejection fraction by MRI, in comparison with other prognostic markers, in patients with PAH. In their study, the authors found that the RV ejection fraction and the right atrium area determined by cardiac MRI both correlated with clinical prognostic markers. However, they showed that the RV ejection fraction showed stronger and more significant correlations than did the right atrium area.
